# A novel online survey approach designed to measure consumer sunscreen application thickness—implications for estimating environmental emissions

**DOI:** 10.1038/s41370-023-00608-z

**Published:** 2023-10-28

**Authors:** Andrea M. Carrao, James C. Coleman, Jeff J. Guo, Harshita Kumari

**Affiliations:** 1 James L. Winkle College of Pharmacy, University of Cincinnati, Cincinnati, OH 45267 USA; 2 Kao USA Inc., Cincinnati, OH USA

**Keywords:** Chemicals in products, Exposure modeling, UV

## Abstract

**Background:**

The effects of ultraviolet (UV) filters in the aquatic environment have been well studied, but environmental exposures remain unclear and understudied. Consumer usage directly influences the amount of sunscreen products, and subsequently UV filters, potentially released into the environment.

**Objective:**

To conduct a literature review of previous research into sunscreen application thickness, develop a questionnaire protocol designed to semi-quantify sunscreen usage by US consumers, and conduct a large-scale survey to determine a sunscreen application thickness (to face and body) that is more refined than conservative defaults. The United States Food & Drug Administration (US FDA) recommends a sunscreen application rate of 2 mg/cm^2^. This value is typically used as a worst-case assumption in environmental exposure assessments of UV filters.

**Methods:**

Designed a novel approach to estimate lotion sunscreen application thickness using an online questionnaire protocol employing visual references and self-reported height and weight of the respondents. A literature review was also conducted to collect historical sunscreen usage.

**Results:**

Over 9000 people were surveyed in the US, and after the dataset was refined, their sunscreen application thickness was estimated based on calculated body surface area and reported sunscreen amounts. The mean and median values for survey respondents are 3.00 and 1.78 mg/cm^2^, respectively, for facial application thickness and 1.52 and 1.35 mg/cm^2^, respectively, for body application thickness. Earlier research from 1985–2020 reported 36 of the 38 values are below the US FDA’s recommended application thickness of 2 mg/cm^2^ (range 0.2–5 mg/cm^2^).

**Impact statement:**

This web-based survey is the first of its kind, designed specifically to quantify sunscreen application in a large and diverse set of consumers. This method provides a greater reach to larger populations thus enabling more granular data analysis and understanding. Exposure assessments of sunscreen ingredients typically use conservative parameters. These data can refine those assessments and allow for more informed and science-based risk management decisions.

## Introduction

Solar ultraviolet (UV) radiation is a known human carcinogen [1]. Sunscreens and other sun protection products protect people from the harmful effects of UV radiation [2] by using organic and inorganic ingredients known as UV filters. UV filters can be used in various combinations and concentrations in skincare product formulations to provide broad-spectrum protection against premature aging and various skin cancers caused by sun exposure. Protection against UV radiation is measured by a numerical sun protection factor (SPF) [3]. While sunscreens play an important role in protecting human health, in recent years there have been numerous scientific and media publications investigating the potential impact of UV filters on environmental health [4–7]. The potential hazard of organic UV filters in the aquatic environment has been well studied, but environmental exposure(s) remain(s) understudied [8].

Consumer habits and practices directly influence the amount of sunscreen and sun protection products, and subsequently UV filters, potentially released into the aquatic environment (i.e.,). Therefore, it is critical to understand consumer use of and preferences for sunscreens and sun protection products when conducting environmental risk assessments (ERA). In the United States, the Food & Drug Administration (US FDA) is responsible for the regulation of all products that claim sun protection under the Over-the-Counter (OTC) Sunscreen Monograph. From this point forward, all sun protection products will be referred to as sunscreens including those that are not designed specifically for use at the beach but instead for daily/routine SPF protection. The US FDA’s standard sunscreen test methods for determining SPF mandate a dermal application of 2.0 milligrams per centimeter squared (mg/cm^2^) [3], which the agency also recommends for consumer use (i.e., application thickness). This value is often used as a default assumption in environmental exposure and risk assessments. However, research over the years indicates that the amount of sunscreen products applied by consumers may be less than the dose used to determine SPF values [real-world application amounts reportedly range from 0.2–1.27 mg/cm^2^] [9–12]. Much of this research determined the application thickness amount by measuring how much of the product was applied by volunteers and the application site’s surface area. Generally, these studies were conducted in specific sub-populations (e.g., skin cancer survivors, beach tourists, etc.). Additionally, there has been little research around routine sun protection habits and practices, including application to the face as part of a daily skincare regimen and the increase in multi-function skincare products with the additional benefit of sun protection (e.g., moisturizing plus SPF). Therefore, new methods are needed to estimate sunscreen application and additional research is needed to determine if previously published application thickness values are representative of the general population and account for more routine use.

In 2022, the National Academies of Sciences, Engineering, and Medicine (NASEM) published the consensus study report *Review of Fate, Exposure, and Effects of Sunscreens in Aquatic Environments and Implications for Sunscreen Usage and Human Health* [13]. This report reviews the state of the science “on the sources and inputs, fate, exposure, and effects of UV filters in aquatic environments, and the availability of data for conducting ERAs.” The report acknowledges that consumer behavior directly affects the environmental exposure of UV filters from sunscreen products. The NASEM report identified several data needs for environmental exposure including amount and type of sunscreen applied, rates of sunscreen application per person, and body coverage of sunscreen.

However, there are several challenges with conducting sunscreen application investigations. They are resource intensive, requiring human subjects and time to conduct studies with an acceptable sample size. Due to the resources required, these studies often target study populations of interest to the investigators. Therefore, alternative methods requiring fewer resources that can be applied to understanding the habits and practices of the general population are still needed. An online platform is one option to reach a larger number of people, increasing the statistical power of the investigation, and allowing more granular analysis of the results. Using this approach, the sunscreen application thickness can be estimated using a visual reference (amount applied) and the volunteers’ disclosed height and weight (skin surface area).

The objective of this research was to develop a web-based survey protocol designed to quantify sunscreen usage by general US consumers, conduct a large-scale survey to determine the application thickness of sunscreen products to participants’ face and body, and perform a literature review of previous research into sunscreen application thickness. Using an online platform to reach a large and diverse sample set, participants were asked about their sunscreen use in general and the amount applied by comparing their use to a visual reference with measured dispensed sunscreen amounts. The desired outcome was to generate a more accurate estimate of dermal application rate of sunscreen products based on current consumer use patterns and preferences that can be used to estimate environmental exposure to UV filters more accurately.

## Methods

### Online survey of sunscreen usage

**Fig. 1:**
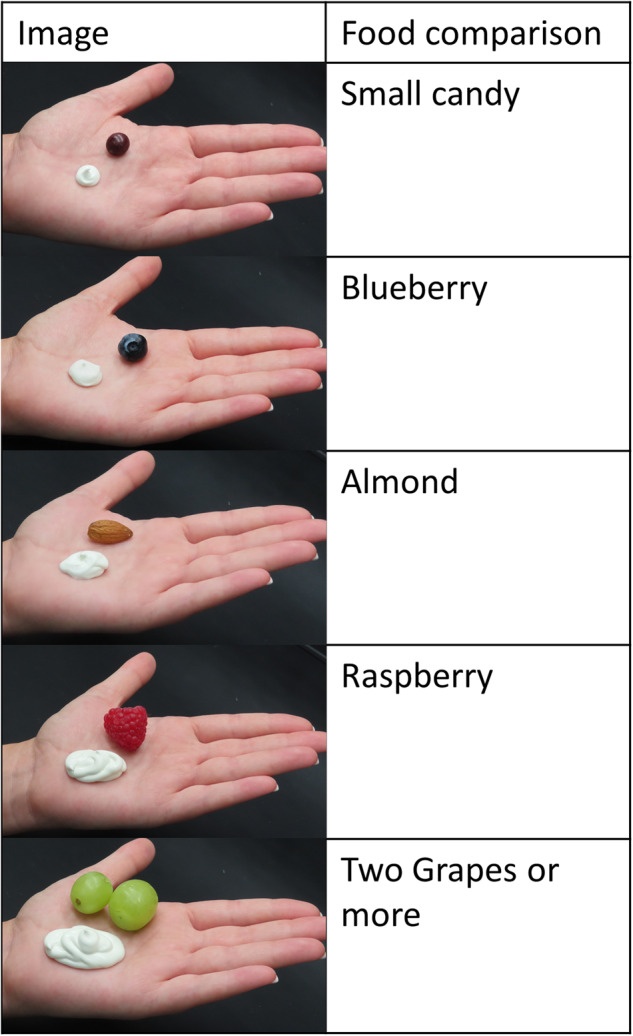
Visual reference used in novel web-based consumer questions. The visual reference includes photos of a measured mass of sunscreen in a hand plus food examples to aid respondents in choosing an estimated sunscreen application amount to their face and both their arms.

An online survey was conducted of the general population in the United States of America. The objective of the survey was to quantify the amount of sunscreen consumers use per sunscreen application. The questionnaire (see Appendix S1) queried participants about their general sunscreen habits, if any, and how much sunscreen they typically apply to the face and both arms. A previous study was conducted using an online survey in Korea to identify common chemicals contained in household and personal care products and how much the respondents use of each product with the goal of conducting an aggregate human exposure assessment [14]. For this research, the survey method was refined with the addition of visual reference photos (Fig. 1) to aid participants in selecting the amount of sunscreen they typically apply per application.

The panel included adults between 18–70 years old that reside in the United States of America. Data from the United States Census Bureau were used to determine quotas for participant genders, ethnicities, ages, and states of residence. Participant ethnicities were included to address possible cultural differences in sunscreen usage among subpopulations. Non-Caucasian ethnicities may be under-studied in sunscreen-related research. To account for this, the present study intentionally over-sampled for African American participants (up to 35%) and balanced the remaining participant ethnicities according to US Census data (see Table S1).

The questionnaire consisted of multiple-choice questions regarding demographic characteristics, sunscreen usage behaviors, reasons for usage, etc. Participants were asked if they applied sunscreen in the last 12 months. If the answer was yes, the participant was directed to a set of sunscreen user questions. If the answer was no, the participant was directed to a set of sunscreen non-user questions.

Candidates were invited to participate in the online self-administered survey hosted by Qualtrics (www.qualtrics.com). Qualtrics uses a mixed-method to recruit individuals. Respondents that previously registered with Qualtrics received a generic email invitation to participate in this study. If the participant agreed to participate, the link in the invitation email directed them to a detailed informed consent form. The participant was asked to review the informed consent and select “agree” to continue with the questionnaire or “disagree” to stop completion of the questionnaire. Double opt-in systems help to ensure data quality by screening out marginally-interested participants. This survey relied on participant self-reporting as the research team had no interaction with them.

After reviewing the results from the initial survey, a second follow-up survey was also conducted with an improved questionnaire. Improvements included refined instructions and reduced survey length to lessen the risk of survey fatigue. The objective of this follow-up survey was to refine the face application thickness estimation due to the observed variance of the initial survey. The follow-up survey was conducted in the United States to test the hypothesis that improved instructions would provide a better-quality (i.e., less varied) dataset. In addition, the instructions for selecting the representative sunscreen amount for application to their face and both their arms had clarified language and pictures added to indicate the application area of interest (see SI Appendix S2). This was done to remove possible ambiguity in the question being asked and clarify that the application site of interest was only the facial area and should not include the participant’s ears, neck, top of head, chest, etc. This survey was sent via SurveyMonkey to the general population but was screened for face sunscreen users, unlike the initial, larger survey. The platform provider was different for this study due to contract changes; however, the questions were programmed so they would be presented to the participants in the same format as the initial study. Two questions were added to qualitatively assess the participants’ facial sunscreen use.

Ethics approval was obtained from the University of Cincinnati Institutional Review Board (IRB) before each survey was initiated (UC IRB #2021-1118 & UC IRB# 2022-0310).

### Application thickness estimation

The data collected from the survey method was used to estimate a single sunscreen application thickness value per participant. The questions related to sunscreen application thickness were narrowed to the face and both arms. Instead of estimating sunscreen application to the entire body, it was thought that it would be easier for participants to separately consider and visualize specific application areas. Habits and practices of face sunscreen use have been changing in the past few years [15]; therefore, face application was one application area of interest. Application to both arms was included as a representative site for body application from the neck down.

The survey questionnaire was used to collect two key data points: (1) the amount of sunscreen typically applied to an individual’s face and both arms using a reference image and (2) each participant’s height and weight. These data were then combined to estimate each participant’s applied sunscreen amount and body surface area (BSA). Taken together, a face and body application thickness can be calculated (mg/cm^2^).

Each participant was asked to review the reference image (Fig. 1) and indicate how much sunscreen they typically apply to their face and to both arms in two separate questions. In addition to a reference amount of sunscreen, common/easily recognizable food items were included in the reference image to further aid participants in estimating and recalling previous sunscreen applications. The amounts used in each picture were weighed prior to the survey and correspond to each food item: small candy = 0.75 g; blueberry = 1.25 g; almond = 2 g; raspberry = 5 g; and two grapes = 10 g. The height (recorded in feet and inches) and weight (recorded in pounds) of each individual participant were noted and were inputted into the BSA calculation from the United States Environmental Protection Agency’s (US EPA) equation [16], BSA = 0.0239 × (H^0.417) × (W^0.517). The resultant BSA value was calculated in centimeter squared for further analysis. The surface area of the participant’s face was estimated to be 5.5% of their total BSA (4.5% face plus 1% accounting for application with fingers) and both arms were estimated to be 18% of the participant’s total BSA [17].

Participants’ body weights were collected in 10-pound increments. The lower value in the weight range was used for the BSA range because this would provide the most conservative estimate of application thickness (i.e., amount applied to a smaller surface area gives a higher mg/cm^2^ estimate). And while some participants might under-report their actual weight due to not being weighed recently or possible societal stigmas, using the lower value in the weight range results in a higher estimated application thickness amount. The conservative nature of the application thickness estimation adds a margin of safety when used in both environmental exposure estimates and possible human health exposure assessments.

### Literature review

A literature review was conducted to serve as a test of validity of the survey results [18] and searched for all previously published studies quantifying sunscreen application thickness. A review of several websites using a set of keywords was used to identify a base set of papers. The initial search was conducted with Science Direct, PubMed, and Google Scholar using a combination of the following keywords: sunscreen application, sunscreen use/usage, consumer sunscreen application rate. Only papers published in English were searched.

Once the base set of papers was curated, inclusion and exclusion criteria were applied to identify the most relevant papers. Only those studies conducted with adults (>18 years old) were included. Studies that included measurements of the sunscreen application thickness to volunteers’ face and body were included. In addition, only studies that used lotion type products were included; therefore, studies measuring application of spray products, make-up, or lip products were excluded from the review. There were no geographic exclusion criteria.

After applying inclusion and exclusion criteria, a snowballing technique [19] was used on the core set of papers. For each paper, the text (forward snowballing) and the reference list (backward snowballing) were reviewed for further research to include in this literature review. Additionally, to ensure inclusion of the greatest number of relevant studies, the most frequently cited papers were selected for additional searching using Connected Papers (https://www.connectedpapers.com/). This website connects publications based on their similarity and allows the identification of additional relevant publications.

Each paper was reviewed, and the reported application thickness amounts were collected along with the year of the study, details of the study population, geographic location, the method of measurement, and the study aim.

### Statistical analysis

After each individual sunscreen application thickness was estimated, a logarithmic multiple variable regression analysis was conducted using IBM SPSS Statistics (version: 28.0.0.0 (190)) software to determine if any of the independent variables were significant predictors of sunscreen application thickness to the face or both arms. For this analysis, the statistical significance level used is 0.05. Non-numeric independent variables were transformed to numeric values (Table S4). Summary statistics were also calculated for each data set using IBM SPSS Statistics.

## Results

The following sections summarize the history of published application thickness values since 1985 and the estimated application thickness values for sunscreen use on the face and body.

### Semi-quantification of sunscreen application thickness to consumer’s face and arms

The questionnaire was in the field January 2022 and after Qualtrics removed incomplete and straight-lined (i.e., same response for each question) responses, a total of 9102 valid participant responses from the United States remained. Nearly 70% of respondents (*n* = 6325 of 9102 total) had used sunscreen at least once in the past 12 months (Table S3). The data from the 6325 respondents that reported sunscreen use in the past 12 months were separated into two datasets: face application thickness and both arms application thickness. For each of these datasets, blank responses for that application site were removed along with any that responded “I typically don’t apply sunscreen to my face” or “I typically don’t apply sunscreen to my arms.” Next, in order to quickly identify incongruent height and weight combinations (e.g., 7’10” and 80 pounds), body mass index (BMI) [20] was estimated for each response and the dataset was sorted smallest to largest. The use of BMI as a filter to the dataset is not used to determine healthiness of the participants and was simply used to refine the current dataset recognizing the possibility of data entry errors. A BMI range of 14–40 (roughly equivalent to the 5th and 95th percentiles) was applied to each dataset as inclusion criteria for values falling within this range, resulting in 5399 responses for face application and 5203 responses for both arms application. The final filter applied to the data was removal of responses from individuals that did not use a lotion product in the last 12 months. The final dataset used to conduct the analysis is comprised of 4338 responses for face application and 3443 responses for both arms application.

The summary statistics for each dataset are listed in Table 1. The mean value for the face application thickness for all respondents is 3.00 mg/cm^2^ and 1.52 mg/cm^2^ for the application thickness of both arms. The median values for face and arm application thickness are 1.78 mg/cm^2^ and 1.35 mg/cm^2^, respectively. The range of values between the two datasets is also quite different. The dataset for face application thickness is highly skewed and has a large amount of variance. The mean and median values for the arm application thickness dataset are much closer in value when compared to the face application thickness value, further illustrating the skewness of the face dataset. Based on the frequency distributions, the median (1.78 mg/cm^2^) of the face application dataset is likely more relevant whereas the mean (1.52 mg/cm^2^) of the arms application dataset is the more relevant value. The application thickness results for both arms are more closely aligned with published values while the face application thickness dataset is quite different. Possible reasons for this difference will be discussed.

**Table 1:** Comparison of summary statistics results for sunscreen application thickness from a large-scale online survey and a follow-up refined survey of the US population.

Application site	Face 1	Both arms 1	Face 2	Both arms 2
Sample size	4338	3443	2192	2020
Range	0.51–15.38 mg/cm^2^	0.15–4.94 mg/cm^2^	0.39–14.33 mg/cm^2^	0.14–4.26 mg/cm^2^
Mean	3.00 mg/cm^2^	1.52 mg/cm^2^	2.78 mg/cm^2^	1.44 mg/cm^2^
Standard deviation	2.9	1.1	2.59	1.06
Median	1.78 mg/cm^2^	1.35 mg/cm^2^	1.70 mg/cm^2^	1.27 mg/cm^2^
Variance	8.385	1.201	6.723	1.127
Skewness	1.79	0.661	1.888	0.641

The initial histograms for application thickness illustrated a significant positive skew (Fig. S3); therefore, the application thickness values for both the face and arms were logarithmically (log_10_) transformed before conducting logarithmic regression and additional statistical analysis. To determine which variables impacted application thickness on the face or arms, a multiple regression analysis of the log transformed values was conducted for each dataset. The variables included: state of residence, gender identity, age range, ethnicity, self-reported skin response to sun exposure (i.e., tendency to burn), Fitzpatrick skin type [21], reported history of skin cancer, the SPF range of the typical sunscreen used, if children are part of the household, use of sunscreen when planning to spend more than 30 min outdoors, use of sunscreen as part of their daily skincare routine, and residence in a warm or cold state (classified based on an average annual temperature from 1901–2000 above (warm) and below (cold) 50 °F [22]). For the log transformed face application thickness variable, the *R*^2^ is 0.030 (*F*(12, 4331) = 11.109, *p* < 0.001) and for the log transformed arms application thickness the *R*^2^ is 0.066 (*F*(12, 3442) = 20.093, *p* < 0.001; Tables S5 and S6). The predictors that had statistical significance for the face application thickness variable were age (*p* = 0.004), ethnicity (*p* = 0.030), reported skin response to sun exposure (*p* < 0.001), reported history of skin cancer (*p* = 0.018), product SPF range used (*p* < 0.001) and the use of sunscreen in a regular skincare routine (*p* < 0.001). The predictors that had statistical significance for both arms application thickness variable were gender identity (*p* < 0.001), ethnicity (*p* = 0.045), Fitzpatrick skin type (*p* < 0.001), reported history of skin cancer (*p* = 0.020), and product SPF range used (*p* < 0.001).

In Table 2, the significance of each independent variable to the dependent variable of application thickness is provided. For facial application thickness, age range has a negative correlation indicating younger sunscreen users will apply more sunscreen. There is a positive correlation of ethnicity to application thickness but no valuable interpretation can be gained from this due to the fact that ethnicity is not a scaled variable. A person’s tendency to burn (skin response to sun exposure) is negatively correlated with thickness application meaning those that tend to burn more will apply a greater amount of sunscreen to their face. The same can be said of those with a self-reported history of skin cancer and those that regularly use a sunscreen product as part of their skincare routine. The product SPF range used by participants is positively correlated to facial application thickness meaning those that use a higher SPF product tend to apply more sunscreen per application. For application to both arms (i.e., body), gender identity is negatively correlated to application thickness suggesting women typically apply a greater amount of sunscreen to their body. Both Fitzpatrick skin type and history of skin cancer are negatively correlated to application thickness. Those with lighter skin tone and/or a history of skin cancer apply a greater amount of sunscreen to their body. Like facial application, product SPF range is positively correlated to application thickness indicating the higher product SPF used then the more sunscreen is generally applied. Again, ethnicity is positively correlated but no interpretation can be made based on these results.

**Table 2:** Significance of variables to the dependent variable of the log transformed sunscreen thickness values.

Dependent variable	Independent variable	Unstandardized *B*	Standardized coefficients *β*	*t*	Significance
Face application thickness (log_10_)	(Constant)	0.414		9.660	<0.001
	State of residence	0.001	0.030	1.948	0.051
	Gender identity	−0.003	−0.009	−0.562	0.574
	Age range	−0.012	−0.054	−2.900	0.004
	Ethnicity	0.006	0.043	2.173	0.030
	Skin response to sun exposure	−0.019	−0.073	−4.385	<0.001
	Fitzpatrick skin type	0.001	0.010	0.524	0.600
	History of skin cancer	−0.034	−0.037	−2.373	0.018
	Product SPF range used	0.029	0.091	5.958	<0.001
	Children in the household	0.011	0.016	0.996	0.319
	Use of sunscreen when outdoors more than 30 min	−0.009	−0.029	−1.467	0.142
	The use of sunscreen in skincare routine	−0.020	−0.067	−3.403	0.001
	Residence in a warm or cold state	−0.004	−0.005	−0.351	0.726
Arms application thickness (log_10_)	(Constant)	0.095		1.875	0.061
	State of residence	0.000	0.017	0.988	0.323
	Gender identity	−0.074	−0.206	−11.881	<0.001
	Age range	0.005	0.023	1.113	0.266
	Ethnicity	0.006	0.044	2.001	0.045
	Skin response to sun exposure	0.008	0.028	1.533	0.125
	Fitzpatrick skin type	−0.009	−0.067	−3.323	<0.001
	History of skin cancer	−0.040	−0.040	−2.329	0.020
	Product SPF range used	0.039	0.114	6.813	<0.001
	Children in the household	0.004	0.005	0.301	0.763
	Use of sunscreen when outdoors more than 30 min	0.000	0.000	−0.021	0.983
	The use of sunscreen in skincare routine	0.001	0.003	0.114	0.909
	Residence in a warm or cold state	0.018	0.022	1.291	0.197

The second survey was in the field during May 2022 and resulted in a sample size of 2192 participants. Though some refinement of the dataset was achieved, the results for the follow-up survey had very similar results as the first study (see Table 1). For facial sunscreen application thickness, the dataset was still highly skewed (skewness = 1.888) and had significant variability (range: 0.39–14.33 mg/cm^2^; variance: 6.723). The second survey asked participants if they apply a greater amount of sunscreen to their face compared to their body (Fig. S4). Participants agreed strongly or somewhat agreed that they apply a greater amount of sunscreen to their face (69%).

### Previously reported application thickness

The literature review search criteria initially identified 43 papers related to sunscreen application. After the inclusion and exclusion criteria was applied (quantified lotion sunscreen application thickness to the body and/or face in adult volunteers), 25 publications measuring sunscreen application thickness were included in the review. Each paper was reviewed, and data were extracted into summary Table S2. It should be noted that a critical review of each study’s method of measuring application thickness was not conducted; instead, results are reported as published. In total, 39 values of application thickness were identified from the 25 studies that were conducted around the world (Fig. S1). Only four studies reported sunscreen application thickness to the face. The majority of studies measured and reported values for the whole body, including the head.

**Fig. 2:**
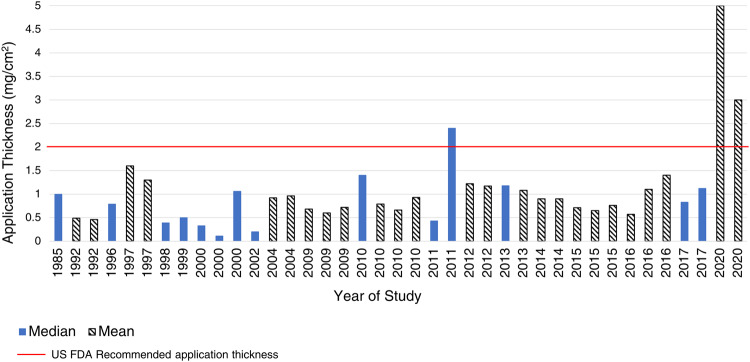
Measured sunscreen application thickness values published since 1985. The figure includes the published application thickness values (mean - blue solid bar, median black and white striped bar) that have been reported in the literature (1985–2020).

All reported values are collated in Fig. 2 and cover the years 1985–2020. The data were not consistently reported in the literature with summary statistics including a mixture of mean and median values for application amounts. For the years 1985–2017, 14 reported median values ranged from 0.2 to 2.4 mg/cm^2^ [11, 23]. For the years 1992–2020, 25 reported mean values ranged from 0.46 to 5 mg/cm^2^ [24, 25]. The red line in Fig. 2 represents the US FDA’s recommended application thickness (2 mg/cm^2^). This figure illustrates how consumers have consistently applied an inadequate amount of sunscreen over the years. Petersen and Wulf [12] conducted a review of sunscreen application thickness and also observed the lower sunscreen application amount versus authority recommendations. They stated, “there is a discrepancy between the amount of sunscreen applied during testing and in reality”. Of note, two values from a 2020 study [24] were above the FDA-recommended application thickness and were obtained from volunteers with a history of skin cancer applying sunscreen to their face. This confounding variable likely accounts for higher use compared to other sub-populations.

The literature review also summarized the methods employed to measure sunscreen application thickness from all the reviewed studies. Five measuring techniques are identified along with their respective percentage use in determining the 39 reported application thickness measurements (Fig. S2). The most common technique was to simply weigh the sunscreen product before and after application to determine the amount applied. Then, the investigators estimated the application surface area using different BSA calculation methods [17, 26, 27]. Additional methods such as tape stripping [11, 28], skin swabbing [29], and fluorescence dose-response [30, 31] have been investigated to determine application thickness but have not been widely adopted based on the results of this literature review.

## Discussion

The results of this research illustrate how a large-scale online consumer survey can be used successfully to collect data for consumer application of sunscreen products. The estimated sunscreen application thickness for both the participants’ arms (mean = 1.52 mg/cm^2^; median = 1.35 mg/cm^2^) is greater than several of the measured values reported in the literature (mean range 0.46–5 mg/cm^2^; median range 0.2–2.4 mg/cm^2^); however, both values are still below the US FDA recommended application thickness of 2 mg/cm^2^. In addition, the observed variability of the application thickness to both arms from this research also reflects a similar range as compared to the historical data set (this study: 0.15–4.94 mg/cm^2^ and literature review results: 0.2–5 mg/cm^2^ [11, 24]). For body estimates of sunscreen application, the method designed for this study is a viable option to reach a large population of sunscreen users.

As stated previously, the US FDA recommended application thickness is typically used in exposure models to account for consumer use and provide some level of conservativeness in the assessment. But to move toward more realistic environmental exposure assessments that can better inform risk management decisions, refinement of UV filter environmental emissions is needed. The NASEM report states “models of the environmental impact of UV filters that rely on currently recommended doses of sunscreen likely overestimate environmental outcomes and would be considered upper bounds [13].” While the US FDA recommended application thickness of 2 mg/cm^2^ does not appear to be vastly different than the current mean value of 1.52 mg/cm^2^ (both arms application thickness) from this study, the significance can be demonstrated with a simple exposure model. For this example Waikiki beach in Honolulu County, Hawaii will be used. In 2021, the beach received 9,284,101 visitors (https://emergencyservices.honolulu.gov/). Using this data, plus several additional assumptions, the total possible sunscreen emission to the aquatic environment can be roughly estimated and compared. Assuming the annual visitation is evenly distributed for each day (25,463 per diem), 70% of the visitors apply sunscreen (this research), 50% of the beach visitors enter the water (conservative assumption), 75% of each person’s body is covered with sunscreen (conservative assumption), 24% [32] of the UV filter is rinsed off the body, and the average body surface area is 18,352.59 cm^2^ (this research), the potential direct release of sunscreen from a single application can be calculated. For the worst-case scenario of the US FDA recommended application of 2 mg/cm^2^, up to 59 kilograms of sunscreen may end up in the environment at Waikiki beach per day. However, using the data from this research, up to 45 kilograms of sunscreen may end up in the environment at Waikiki beach per day, nearly 24% less than the upper bound value. Comparing these two results illustrates the value of refining conservative emissions assessments and the relevance of consumer sunscreen usage research. There are two important points to note. First, these are very conservative assumptions used for illustrative purposes only and are not assessments that should be used in any type of environmental risk assessment or to inform risk management decisions. This example does not include the environmental fate of the target chemicals nor any type of degradation. Second, this is total sunscreen mass from a single application and does not account for the UV filter formula composition or differences in reapplication thickness.

Further refinement and consumer research is needed to make this method more reliable for estimated sunscreen application thickness to the face. The dataset for face application thickness is positively skewed (skewness: 1.79) and has a large amount of variability (range: 0.52–15.38 mg/cm^2^; variance: 8.385). The sunscreen market is growing and evolving beyond only sunscreen products designed for use at the beach. The market now includes multi-functional products designed with SPF protection and products designed for daily sun protection [33]. The survey conducted for this research did not distinguish between lotion products designed for beach use (traditional sunscreen) and those designed for daily/routine sun protection when asking participants about their sunscreen application amounts. With this change in consumer habits and practices since the US FDA OTC Sunscreen monograph was published in 1972, further studies are needed to understand both the frequency of use and the amount of sunscreen applied and reapplied to the face.

The results of the literature review indicate that consumers historically have not applied adequate amounts of sunscreen lotion to achieve the labeled sun protection factor. And while the general trend is toward increasing application amounts over time, there is not a way to measure the significance of the trend due to the differences in the reported measurement methods and data analysis. Anecdotally, this increasing trend may be evidenced in the rising prominence of online skincare influencers and the continued growing sunscreen market [15].

Due to the COVID pandemic, survey participants may not be going to the beach as frequently as they typically would and may not have an accurate recollection of their typical sunscreen use. Similarly, these questionnaires rely on self-reporting which has limits on obtaining the most accurate data. Also, the results from this research are semi-quantitative and not an exact measure of sunscreen application amounts which limits the distribution of the results. Respondents may have also misread the application amount question and answered it as the amount they apply for the entire day instead of a single application. The combination of these conditions may lead to a greater amount of uncertainty compared to more controlled sunscreen application studies that have been conducted in the past. Nonetheless, results in this study are supported by previous work showing similar trends for body application thickness.

Despite the potential increased uncertainty in the data, they still provide new value when attempting to determine the impacts of different UV filters on the environment and human health. For sunscreen application to the body, the current research dataset and the historical data reveal consumers are not applying the recommended amount of sunscreen. Therefore, using 2 mg/cm^2^ as an assumed application thickness in any UV filter exposure and/or risk assessment (such as the maximal usage trial (MUsT) as executed and required by US FDA [34, 35]) is likely to yield an overestimate of exposure. Therefore, data and insights from this research can be used to ground truth current human health exposure and risk assessments related to UV filters and other sunscreen ingredients for both beach and routine daily sun protection products. Employing more realistic sunscreen usage estimates can also better inform co-exposure assessments since many sunscreens contain more than one UV filter per product and UV filters are found in other personal care products.

These insights can also be used to improve recommendations and education campaigns around safe sun exposure. Skin cancer is the most diagnosed cancer in the US [13]. In 2019, the incidence of skin cancer was six times higher than it was 40 years ago, which is out of proportion when compared to other types of preventable cancers [13]. In 2013, 39.5 million Americans sought medical care due to sun-related skin damage resulting in a cost of $1.8 billion [36]. The NASEM report found the “consistent use of broad spectrum, SPF 30 sunscreen when outdoors reduces the risk of developing skin cancer (keratinocyte carcinomas and melanomas), photoaging, and sunburn.” As this research illustrates, many people are not applying adequate sunscreen to ensure protection from the harmful effects of chronic sun exposure and the burden of these effects has been increasing over the years. Thus, when conducting environmental risk assessments of UV filters, the importance of sunscreen use to human health cannot be ignored. Replacing overly conservative assumptions with more accurate value that represent current consumer sunscreen use will result in more realistic exposure and risk assessments and lead to better informed and balanced risk management actions for both human and environmental health.

The survey and questionnaires for this research were specifically designed to test the use of an online platform to estimate sunscreen application thickness. The results illustrate the success of this method. However, the current study design needs additional refinement to clarify specific independent variables that predict sunscreen application thickness and to what extent the variables influence the application thickness of different sub-populations. Furthermore, the visual reference should be updated for facial sunscreen application. Using such large amounts for a small area of the body may contribute to the large range and possible misunderstanding on the part of the participants. Further research is also needed to develop a better understanding of consumer facial and body sunscreen reapplication thickness and frequency.

In the end, this study demonstrates that there are many factors influencing an individual’s sunscreen usage. The results of this study confirm that the general population does not apply the recommended amount of sunscreen to the body. Consumers who apply sunscreen to their face apply a greater amount than previously anticipated. These data can be used to refine risk assessments of UV filters applied to the body and directly enter the environment at the beach, but further work is needed to improve ERAs for UV filters in facial sunscreen products.

## Supplementary information

Supplementary Material

Supplemental Information - Sunscreen Survey Data

Reporting Checklist

## Data Availability

All data used in this study analysis are available as a Microsoft Excel file in the Supplementary Information accompanying this paper.
