# Supplementary material for: A novel online survey approach designed to measure consumer sunscreen application thickness—implications for estimating environmental emissions
Source: J Expo Sci Environ Epidemiol. 2023 Oct 28;34(6):1064–71. doi: 10.1038/s41370-023-00608-z (PMC11618062; doi:10.1038/s41370-023-00608-z)
Supplement: Supplementary file 1 — Supplementary Material [file 41370_2023_608_MOESM1_ESM.docx]

**Supplementary Material**

**Title:** *A Novel Online Survey Approach Designed to Measure Consumer Sunscreen Application Thickness – Implications for Estimating Environmental Emissions*

**Authors**: Andrea M. Carrao^a,b,*^, James C. Coleman II^b^, Harshita Kumari^a^

^a^James L. Winkle College of Pharmacy, University of Cincinnati, Cincinnati, OH

^b^Kao USA Inc., Cincinnati, OH

*Corresponding author

**Contents:**

Table S1: Sample set quotas

Table S2: Detailed List of Literature Results

Figure S1: Map of Countries Were Sunscreen Application Thickness Investigations were Conducted

Figure S2: Sunscreen application thickness measuring techniques reported in the literature (1985-2020).

Appendix 1: Questionnaires

- Questionnaire S1: Initial Online Consumer Survey Questionnaire
- Questionnaire S2: Follow-up Face Consumer Survey Questionnaire

Table S3: US reported sunscreen use in the last 12 months by gender, ethnicity, age, skin reaction to sun, Fitzpatrick skin type, and reported history of skin cancer

Figure S3: Histograms of Application Thickness values – both untransformed and log_10_ transformed

Table S4: Numeric assignments to independent variables

Table S5: Logarithmic multiple variable regression analysis model results for sunscreen application thickness dataset

Table S6: ANOVA table for sunscreen application thickness from an online survey of the US general population

Figure S4: Respondents answers to the question “How much do you agree or disagree with the following statements:

References

**Table S1: Sample set target quotas for initial large-scale survey**

Target N = 9,000

Age 18-70

Gender

Male: 48%

Female: 52%

Non-binary: natural fallout

Age

Age 18-34: 30%

Age 35-54: 32%

Age 55+: 38%

Ethnicity:

Non-Hispanic White: 39.5%

Non-Hispanic Black: 35% (+10% over quota)

Hispanic: 18.5%

Other: 8%

| State | 2020 Census | Percent of Total | Target range |
| --- | --- | --- | --- |
| California | 39,538,223 | 11.91% | 10-15% |
| Texas | 29,145,505 | 8.74% | 5-10% |
| Florida | 21,538,187 | 6.47% | 5-10% |
| New York | 20,201,249 | 5.86% | 5-10% |
| Pennsylvania | 13,002,700 | 3.86% | 1-5% |
| Illinois | 12,801,989 | 3.82% | 1-5% |
| Ohio | 11,799,448 | 3.52% | 1-5% |
| Georgia | 10,711,908 | 3.20% | 1-5% |
| North Carolina | 10,439,388 | 3.16% | 1-5% |
| Michigan | 10,077,331 | 3.01% | 1-5% |
| New Jersey | 9,288,994 | 2.68% | 1-5% |
| Virginia | 8,631,393 | 2.57% | 1-5% |
| Washington | 7,705,281 | 2.29% | 1-5% |
| Arizona | 7,151,502 | 2.19% | 1-5% |
| Massachusetts | 7,029,917 | 2.09% | 1-5% |
| Tennessee | 6,910,840 | 2.06% | 1-5% |
| Indiana | 6,785,528 | 2.03% | 1-5% |
| Maryland | 6,177,224 | 1.85% | 1-5% |
| Missouri | 6,154,913 | 1.82% | 1-5% |
| Wisconsin | 5,893,718 | 1.75% | 1-5% |
| Colorado | 5,773,714 | 1.74% | 1-5% |
| Minnesota | 5,706,494 | 1.70% | 1-5% |
| South Carolina | 5,118,425 | 1.55% | 1-5% |
| Alabama | 5,024,279 | 1.48% | 1-5% |
| Louisiana | 4,657,757 | 1.40% | 1-5% |
| Kentucky | 4,505,836 | 1.35% | 1-5% |
| Oregon | 4,237,256 | 1.27% | 1-5% |
| Oklahoma | 3,959,353 | 1.19% | 1-5% |
| Connecticut | 3,605,944 | 1.07% | 1-5% |
| Utah | 3,205,958 | 0.97% | <1% |
| Iowa | 3,271,616 | 0.95% | <1% |
| Nevada | 3,104,614 | 0.93% | <1% |
| Arkansas | 3,011,524 | 0.91% | <1% |
| Mississippi | 2,961,279 | 0.90% | <1% |
| Kansas | 2,937,880 | 0.88% | <1% |
| New Mexico | 2,117,522 | 0.63% | <1% |
| Nebraska | 1,961,504 | 0.58% | <1% |
| Idaho | 1,839,106 | 0.54% | <1% |
| West Virginia | 1,793,716 | 0.54% | <1% |
| Hawaii | 1,455,271 | 0.43% | 85 responses |
| New Hampshire | 1,377,529 | 0.41% | <1% |
| Maine | 1,362,359 | 0.41% | <1% |
| Rhode Island | 1,097,379 | 0.32% | <1% |
| Montana | 1,084,225 | 0.32% | <1% |
| Delaware | 989,948 | 0.29% | <1% |
| South Dakota | 886,667 | 0.27% | <1% |
| North Dakota | 779,094 | 0.23% | <1% |
| Alaska | 733,391 | 0.22% | <1% |
| DC | 689,545 | 0.21% | <1% |
| Vermont | 643,077 | 0.19% | <1% |
| Wyoming | 576,851 | 0.17% | <1% |

**Table S2: Detailed List of Literature Results**

| **Reference** | **Location** | **Study year** | **Number of participants** | **Application thickness** | | **Study method** |
| --- | --- | --- | --- | --- | --- | --- |
|  |  |  |  | **Median** | **Mean** |  |
| Stenberg et al, 1985 | Sweden | not reported | 50 – body  (42 female; 8 male;  age range: 35-61 years) | ***~1 mg/cm^2^*** |  | Applying & weighing products in a lab then dividing by measured area of application |
| Bech-Thomsen et al, 1992 | Denmark | 1992 | 32 – body (16 female; 16 male;  age range: not reported) |  | ***0.46 mg/cm^2^*** - women ***0.49 mg/cm^2^*** - men | Applying & weighing products at the beach and extrapolating to estimated skin surface area |
| Neale et al, 2002 | Australia | 1992 to 1996 | 764 – body  (428 female; 336 male;  mean age: 49 years) | ***0.79 mg/cm^2^*** |  | Applying & weighing products and extrapolating to estimated skin surface area |
| Gottlieb et al, 1997 | United States | not reported | 20 – face & body  (17 female; 3 male;  age range: not reported) |  | ***1.6 mg/cm^2^*** - face ***1.3 mg/cm^2^*** - body | Applying & weighing products in a lab and extrapolating to estimated skin surface area |
| Autier et al, 2001 | France, Switzerland & Belgium | 1997 & 1998 | 124 – whole body (84 female; 40 male;  age range: 18-24 years) | ***0.39 mg/cm^2^*** |  | Applying & weighing products and extrapolating to estimated skin surface area |
| Azurdia et al, 1999 | United Kingdom | not reported | 10 – whole body (all female;  age range: 22-73 years) | ***0.5 mg/cm^2^*** |  | fluorescence measurements converted to application thickness via dose-response in a laboratory setting |
| Azurdia et al, 2000 | United Kingdom | not reported | 6 – face & body (5 female; 1 male;  age range: 35-61 years) | ***0.33 mg/cm^2^*** - face  ***0.11 mg/cm^2^*** - body |  | fluorescence measurements converted to application thickness via dose-response in a laboratory setting |
| Hart et al, 2000 | United Kingdom | not reported | 50 – body (arms) (27 female; 23 male;  age range: 16-77 years) | ***1.06 mg/cm^2^*** |  | Applying an analogous cream product & weighing products then measuring skin surface area in a laboratory setting |
| Lademann et al, 2004 | Germany | 2002 | 60 – whole body (27 female; 33 male;  age range: 17-68 years) | ***0.2 mg/cm^2^*** (<10% of 2 mg/cm^2^) |  | Tape stripping skin sites of random beach attendees and extrapolating based on sunscreen concentration |
| Szepietowski et al, 2004 | Poland | not reported | 49 – whole body (24 female; 25 male;  age range: 18-32 years) |  | ***0.96 mg/cm^2^*** - cream ***0.92 mg/cm^2^*** - lotion | Applying & weighing products and extrapolating to estimated skin surface area (Isaksson formula) |
| Reich et al, 2009 | Poland | not reported | 52 – whole body (36 female; 16 male;  age range: 18-68 years) |  | ***0.68 mg/cm^2^*** - all ***0.72 mg/cm^2^*** - women ***0.60 mg/cm2*** - men | Applying & weighing products in a laboratory setting and extrapolating to estimated skin surface area (Isaksson formula) |
| Bauer et al, 2010 | Australia | not reported | 18 – body (arms) (details not reported) | ***1.4 mg/cm^2^*** |  | Skin swabbing method and spectrophotometric analysis |
| Petersen et al, 2013 | Egypt | 2010 | 20 – whole body (10 female; 10 male;  age range: 20-63 years) |  | ***0.79 mg/cm^2^*** - all ***0.66 mg/cm^2^*** - women ***0.93 mg/cm^2^*** - men | Applying & weighing products at a beach setting and extrapolating to estimated skin surface area (Mosteller formula) |
| De Villa et al, 2011 | Brazil | not reported | 36 – body (arms) (21 female; 15 male;  age range: 18-31 years) | ***0.43 mg/cm^2^*** |  | Applying sunscreen in a laboratory setting, tape stripping skin sites and analyzing via HPLC 30 minutes after application |
| Narbutt et al, 2019 | Spain | 2011 | 40 – whole body (21 female; 19 male;  age range: 18+ years) | ***2.4 mg/cm^2^*** |  | Applying & weighing products at the beach and dividing by assumed application surface area of 85% |
| Teramura et al, 2012 | Japan | not reported | 23 – face & body (arms) (6 female; 17 male;  age range: 26-53 years) |  | ***1.17 mg/cm^2^*** - face ***1.22 mg/cm^2^*** - forearm | Applying & weighing products in a laboratory setting, before and after use (BSA determination not reported) |
| Pissavini et al, 2013 | United Kingdom | not reported | 10 – body (arms) (details not reported) | ***1.18 mg/cm^2^*** |  | Weightng of product and applying to a set application area in a lab |
| Novick et al, 2015 | United States | 2013 | 52 – body (arms) (25 female; 19 male; 8 NR  age range: 18+ years) |  | ***1.08 mg/cm^2^*** - lotion | Applying & weighing of products at the beach and extrapolating to estimated skin surface area |
| Ficheux et al, 2016 | France | 2014 | 89 – whole body  (58 female; 31 male  age range: 15+ years) |  | ***0.9 mg/cm^2^*** - women ***0.9 mg/cm^2^*** - men | Applying & weighing of products in a lab and extrapolating to estimated skin surface area |
| Heerfordt et al, 2018 | Denmark | 2015 | 31 – whole body (15 female; 16 male;  age range: 19-40 years) |  | ***0.71 mg/cm^2^*** - all ***0.65 mg/cm^2^*** - women ***0.76 mg/cm^2^*** - men | Applying & weighing of products in a lab and extrapolating to estimated skin surface area (Mosteller formula) |
| Heerfordt et al, 2017 | Denmark | 2016 | 111 – whole body (85 female; 26 male;  age range: 15+ years) |  | ***0.57 mg/cm^2^*** | Applying & weighing of products at the beach and extrapolating to estimated skin surface area (Mosteller formula) |
| Harben et al, 2019 | United States | 2016 | 94 – body (arms & legs) (56 female; 38 male;  age mean: 23 years) |  | ***1.1 mg/cm^2^*** - pump bottle ***1.4 mg/cm^2^*** - squeeze bottle | Applying & weighing of products in a lab and extrapolating to estimated skin surface area (Rule of 9s) |
| Heerfordt et al, 2018 | Denmark | 2017 | 31 – whole body (15 female; 16 male;  age range: 19-40 years) | ***0.83 mg/cm^2^*** |  | Applying & weighing of products in the lab and dividing by measured area of application |
| Heerfordt et al, 2020 | Denmark | 2017 | 17 – whole body  (9 female; 8 male;  age range: 21-42 years) | ***1.12 mg/cm^2^*** |  | Applying & weighing of products in a lab and dividing by measured area of application |
| Dang et al, 2020 | United States | not reported | 70 - face (43 female; 27 male;  mean age: 67.8 years) |  | ***3.0 mg/cm^2^*** - face, women ***5.0 mg/cm^2^*** - face, men | Applying & weighing of products in a laboratory setting before and after use and dividing by median facial surface area |

**Figure S1: Map of Countries Where Sunscreen Application Thickness Investigations were Conducted**


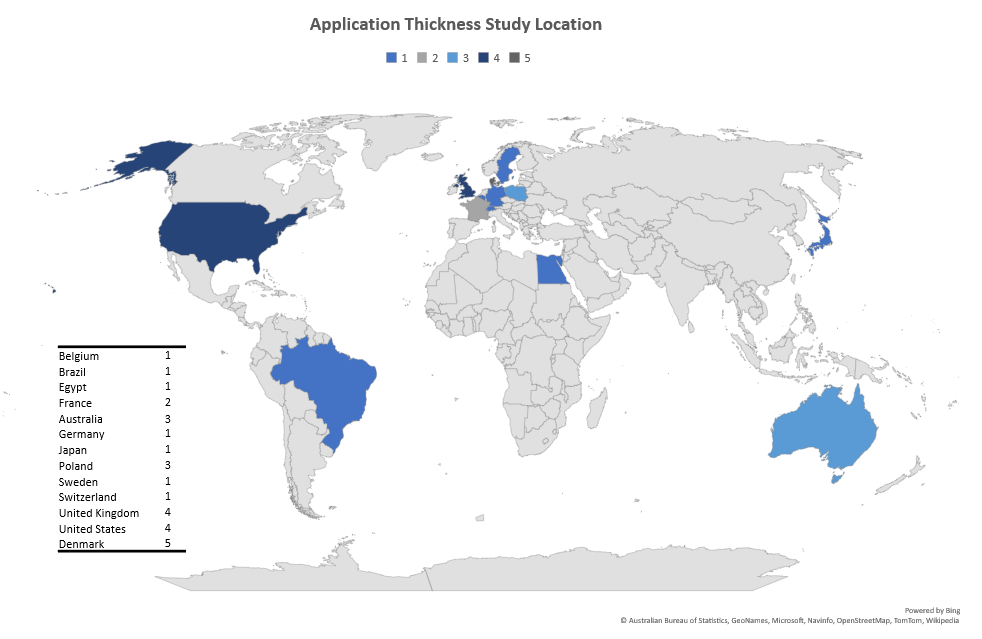


**Figure S2: Sunscreen application thickness measuring techniques reported in the literature (1985-2020).**

**
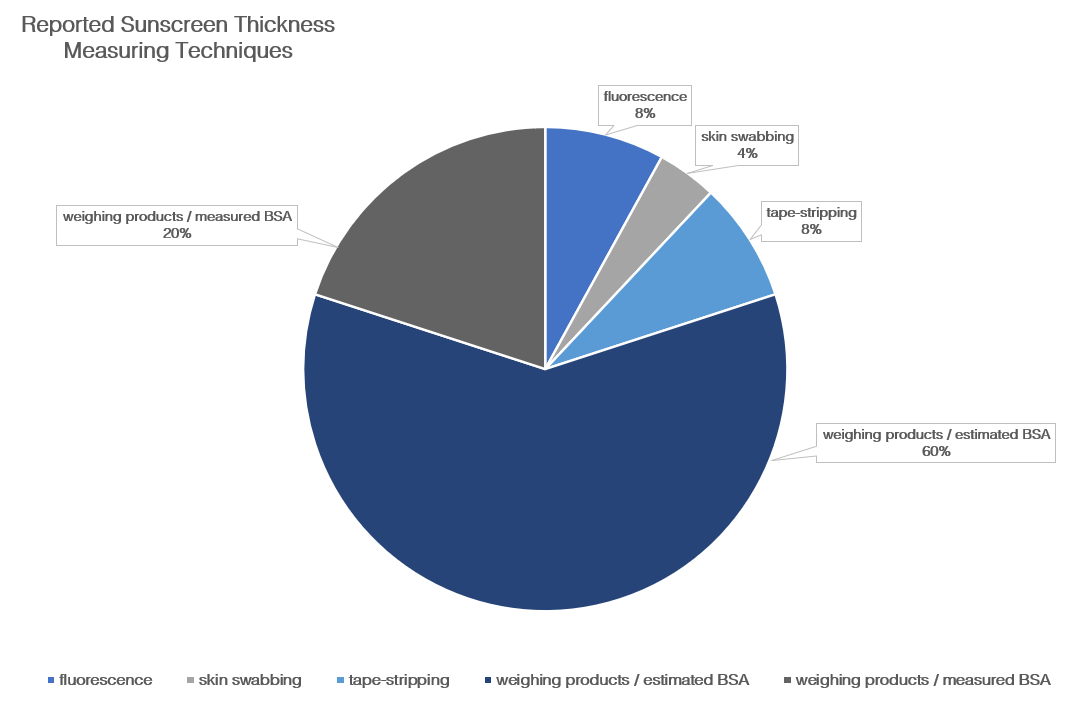
**

**Appendix 1: Questionnaires**

**S1: Initial Online Consumer Sunscreen Survey Questionnaire**

Please provide the US state and zip code where you primarily reside in a typical year.

- State ________________________________________________
- Zip Code ________________________________________________

How do you identify?

- Female
- Male
- Gender variant/non-conforming
- Transwoman (MtF)
- Transman (FtM)
- There’s not a name for it here
- Prefer to self describe ________________________________________________

Which age group are you?

- Under 18
- 18-24
- 25-34
- 35-44
- 45-54
- 55-64
- 65-70
- 71 or older

To make sure that we have a fair representation of consumers, please indicate which of the following best describes the ethnic background you identify with.

- White/Caucasian
- Black/African American
- Asian or Pacific Islander
- Hispanic
- Indigenous
- Bi-racial/Multiple ethnicities
- Other
- Prefer not to answer

Please select the weight in pounds below that is closest to your current weight.

General weight bracket

10 pound increments

▼ 100 pounds or less ... 400 pounds or more ~ 400 pounds or more

Please indicate your height in feet and inches.

Feet

Inches

▼ 4 feet ... 6 feet ~ 11 inch

Now we are going to ask you some questions about products you use to protect yourself from the sun.  For the purposes of this survey, we’d like you to think about those products as five separate categories:

General Body Sunscreen: Products you find in the sun care aisle of the store that are designed for sun protection anywhere on your body.

Sunscreen Designed for the Face: Products you find in the sun care aisle of the store that are specifically designed to provide sun protection for your face.

Daily Face Care Product with SPF: Products you find in the facial care aisle of the store that contain SPF (such as facial moisturizers with SPF, etc.).

Daily Body Care Product with SPF: Products you find in the body and skin care aisle of the store that contain SPF (such as body moisturizers with SPF, etc.).

Cosmetic Product with SPF: Products you find in the cosmetics aisle of the store that contain SPF (such as face foundation with SPF, etc.).

Please indicate which, if any, of the following products have you used in the past 12 months, on your face and/or body - along with the typical SPF level of each product you have used. 
Please select all that apply.

|  | SPF 15 or below | SPF 16-29 | SPF 30-49 | SPF 50+ | Have not used in past 12 months |
| --- | --- | --- | --- | --- | --- |
| Daily face care products with SPF |  |  |  |  |  |
| Daily body care products with SPF |  |  |  |  |  |
| Sunscreen designed for the face |  |  |  |  |  |
| General body sunscreen |  |  |  |  |  |
| Cosmetic product(s) with SPF |  |  |  |  |  |

Now thinking about the product types you typically use, indicate the product form that applies MOST OFTEN to the SPF products you have used in the last 12 months.

|  | Lotion, cream, liquid, milk, serum, gel | Pump or aerosol spray | Powder, stick, roll-on, towelette | Other |
| --- | --- | --- | --- | --- |
| Daily face care products with SPF |  |  |  |  |
| Daily body care products with SPF |  |  |  |  |
| Sunscreen designed for the face |  |  |  |  |
| General body sunscreen |  |  |  |  |
| Cosmetic product(s) with SPF |  |  |  |  |

Which of the following statements best describes your skin when typically exposed to the sun?  Please select one response.

- My skin always burns in the sun, never tans and skin may peel.
- My skin may tan but with difficulty in the sun and my skin may peel.
- My skin burns moderately in the sun and tans gradually.
- My skin rarely burns in the sun and tans easily.
- My skin very rarely burns in the sun and tans very easily.
- My skin never burns in the sun.

Is there any history of skin cancer, for yourself or your family?

- Yes
- No
- N/A
- Don’t know
- Prefer not to answer

Thinking about your skin color or tone, on a scale of 1-12 where a 1 is very fair or pale and a 12 is very dark, what number would you consider your **natural** skin color or tone (without a

suntan or tanning products)?*Please choose the number that most closely represents your skin tone.*


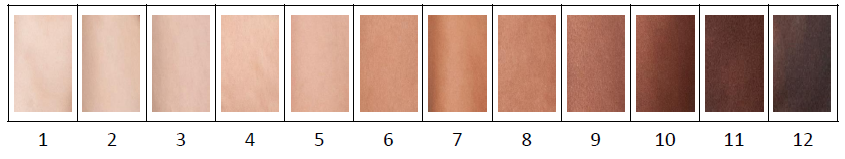


- 1
- 2
- 3
- 4
- 5
- 6
- 7
- 8
- 9
- 10
- 11
- 12

Looking at the images below and considering your typical day at the beach/gulf/ocean or lakes/rivers, how much lotion/cream/gel sunscreen do you typically dispense to apply to your face? Select the most representative amount to the best of your ability. For this question, please disregard other product types (e.g., spray, stick, etc.) and answer only based on the lotion/cream/gel product you last used.

-
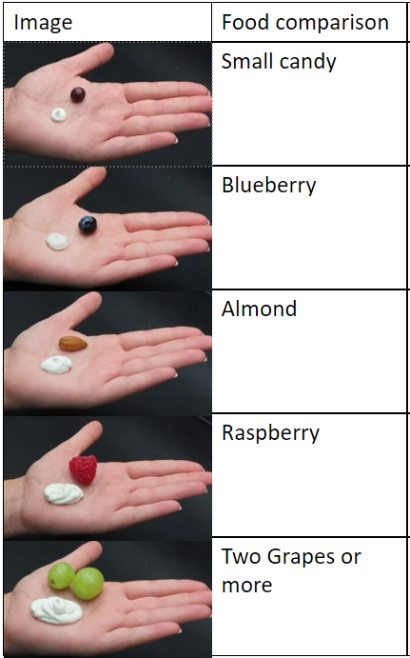
I don't typically apply sunscreen products to my face
- Small candy
- Blueberry
- Almond
- Raspberry
- Two grapes or more

Looking at the images below and considering your typical day at the beach/gulf/ocean or lakes/rivers, how much lotion/cream/gel sunscreen do you typically dispense to apply to **both of your arms**? Select the most representative amount to the best of your ability. For this question, please disregard other product types (e.g., spray, stick, etc.) and answer only based on the lotion/cream/gel product you last used.

-
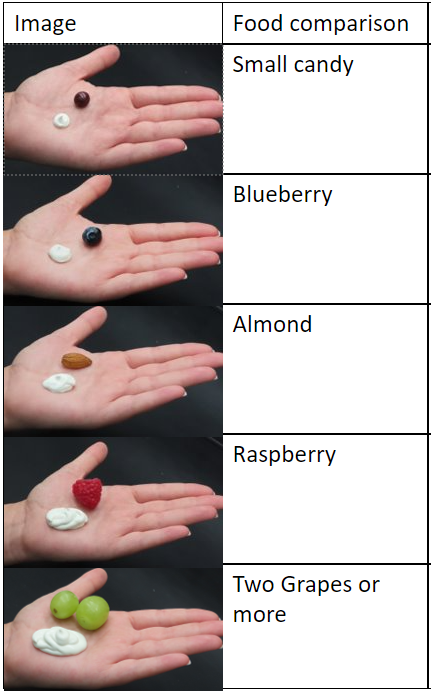
I don't typically apply sunscreen products to my arms
- Small candy
- Blueberry
- Almond
- Raspberry
- Two grapes or more

**S2: Follow-up Sunscreen Face Consumer Survey Questionnaire**

Screener Question:

Have you used and applied a **lotion** SPF product and/or sunscreen to your face in the last 12 months? EXCLUDING COSMETICS (e.g., foundation, powder, primer, etc.)

- Yes
- No

>if answer no, terminate study<

Please provide the US state and zip code where you primarily reside in a typical year.

- State ________________________________________________
- Zip Code ________________________________________________

How do you identify?

- Female
- Male
- Gender variant/non-conforming
- Transwoman (MtF)
- Transman (FtM)
- There’s not a name for it here
- Prefer to self describe _____

Which age group are you?

- Under 18
- 18-24
- 25-34
- 35-44
- 45-54
- 55-64
- 65-70
- 71 or older

To make sure that we have a fair representation of consumers, please indicate which of the following best describes the ethnic background you identify with.

- White/Caucasian
- Black/African American
- Asian or Pacific Islander
- Hispanic
- Indigenous
- Bi-racial/Multiple ethnicities
- Other
- Prefer not to answer

Please select the weight in pounds below that is closest to your current weight.

General weight bracket

10 pound increments

▼ 100 pounds or less ... 400 pounds or more ~ 400 pounds or more

Please indicate your height in feet and inches.

Feet

Inches

▼ 4 feet ... 6 feet ~ 11 inch

Which of the following statements best describes your skin when typically exposed to the sun?  Please select one response.

- My skin always burns in the sun, never tans and skin may peel.
- My skin may tan but with difficulty in the sun and my skin may peel.
- My skin burns moderately in the sun and tans gradually.
- My skin rarely burns in the sun and tans easily.
- My skin very rarely burns in the sun and tans very easily.
- My skin never burns in the sun.

Is there any history of skin cancer, for yourself or your family?

- Yes
- No
- Don’t know
- Prefer not to answer

Thinking about your skin color or tone, on a scale of 1-12 where a 1 is very fair or pale and a 12 is very dark, what number would you consider your **natural** skin color or tone (without a

suntan or tanning products)?*Please choose the number that most closely represents your skin tone.*


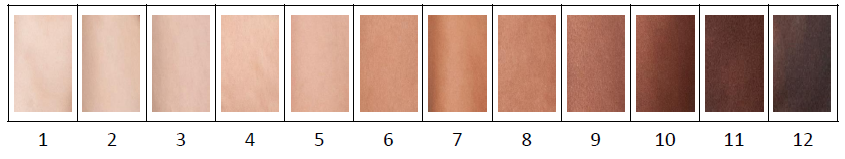


- 1
- 2
- 3
- 4
- 5
- 6
- 7
- 8
- 9
- 10
- 11
- 12

How much do you agree or disagree with the following statement: *I apply more (i.e. greater amount) sun protection product to my face compared to my body.*

- Agree strongly
- Agree somewhat
- Neither agree nor disagree
- Disagree somewhat
- Disagree strongly

How much do you agree or disagree with the following statement: *I apply sun protection product to my face more often (i.e. more frequently) compared to my body.*

- Agree strongly
- Agree somewhat
- Neither agree nor disagree
- Disagree somewhat
- Disagree strongly

For the next two questions we want to learn about how much sun protection product you apply to your face and/or arms. When considering your answer, try to visualize the amount of product you dispense into your hand and apply to your face or body.

Looking at the images below and considering your typical day at the beach/gulf/ocean or lakes/rivers, how much **lotion/cream/gel** sunscreen do you typically dispense to apply to your **face?**

When considering your face, please do not include amount applied to your neck, ears, top of head, etc. – please consider the amount of product applied to your facial area only (see green area in below face diagram).

Select the most representative amount to the best of your ability. For this question, please disregard other product types (e.g., spray, stick, etc.) and answer only based on the lotion/cream/gel product you last used.


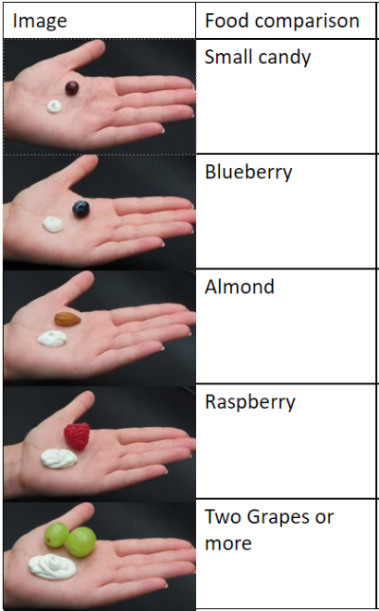

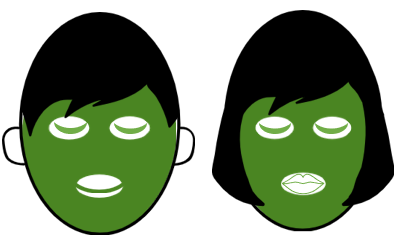


- Small candy
- Blueberry
- Almond
- Raspberry
- Two grapes or more

Looking at the images below and considering your typical day at the beach/gulf/ocean or lakes/rivers, how much **lotion/cream/gel** sunscreen do you typically dispense to apply to **both sides of each of your arms,** including your hands (see green area of arm diagram)?

Select the most representative amount to the best of your ability. For this question, please disregard other product types (e.g., spray, stick, etc.) and answer only based on the lotion/cream/gel product you last used.


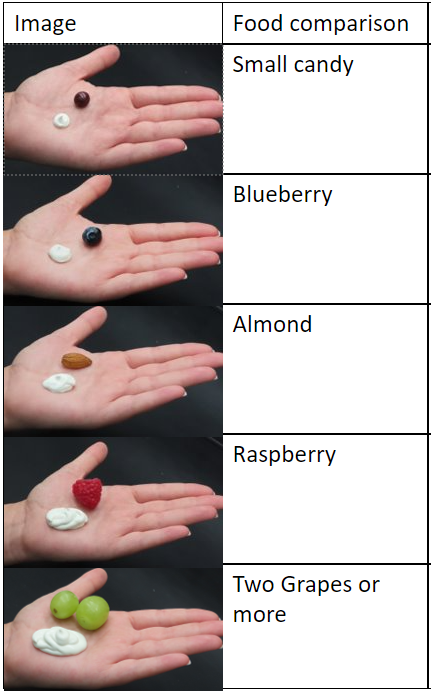

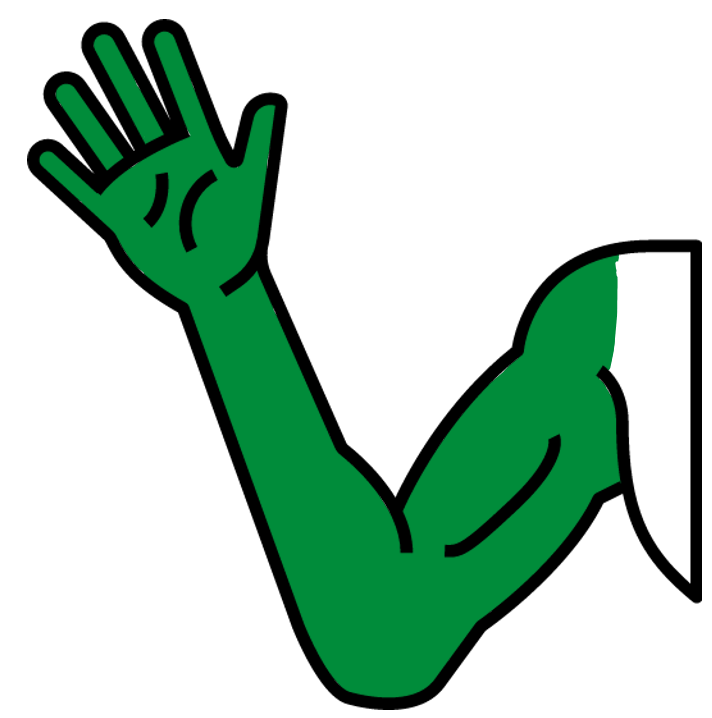

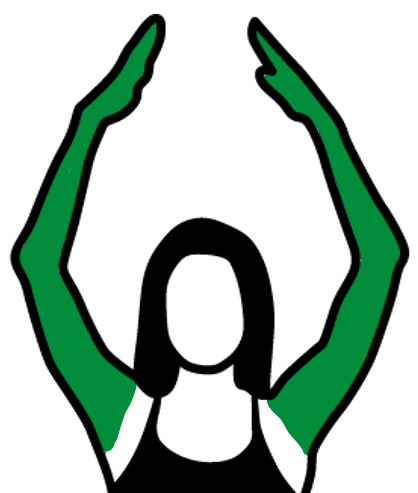


- I don't typically apply sunscreen products to my arms
- Small candy
- Blueberry
- Almond
- Raspberry
- Two grapes or more

**Table S3: US reported sunscreen use in the last 12 months by gender, ethnicity, age, skin reaction to sun, Fitzpatrick skin type, and reported history of skin cancer**

|  |  |  | Have used sunscreen in last 12 months? | |
| --- | --- | --- | --- | --- |
| Category |  | n (%) | Yes | No |
| Total | United States | 9,102 (100) | 69.50% | 30.50% |
|  |  |  |  |  |
| Gender | Female | 4,737 (52.0%) | 73.80% | 26.20% |
|  | Male | 4,303 (47.3%) | 64.70% | 35.30% |
|  | Gender variant/non-conforming | 29 (0.32%) | 62.00% | 38.00% |
|  | Transwoman (MtF) | 6 (0.07%) | 66.70% | 33.30% |
|  | Transman (FtM) | 15 (0.16%) | 80.00% | 20.00% |
|  | There’s not a name for it here | 8 (0.09%) | 62.50% | 37.50% |
|  | Prefer to self describe | 4 (0.04%) | 100% | 0% |
|  |  |  |  |  |
| Ethnicity | Asian or Pacific Islander | 441 (4.8%) | 87.10% | 12.90% |
|  | Bi-racial/Multiple ethnicities | 166 (1.8%) | 65.10% | 34.90% |
|  | Black/African American | 3,111 (34.2%) | 64.90% | 35.10% |
|  | Hispanic | 1,459 (16.0%) | 79.60% | 20.40% |
|  | Indigenous | 56 (0.62%) | 73.20% | 26.80% |
|  | White/Caucasian | 3,796 (41.7%) | 67.80% | 32.20% |
|  | Other | 52 (0.57%) | 61.50% | 38.50% |
|  | Prefer not to answer | 21 (0.23%) | 38.10% | 61.90% |
|  |  |  |  |  |
| Age | 18-24 | 1,224 (13.4%) | 76.06% | 23.94% |
|  | 25-34 | 1,509 (16.6%) | 80.32% | 19.68% |
|  | 35-44 | 1,757 (19.3%) | 77.23% | 22.77% |
|  | 45-54 | 1,156 (12.74%) | 65.74% | 24.26% |
|  | 55-64 | 2,008 (22.1%) | 62.75% | 37.25% |
|  | 65-70 | 1,448 (15.9%) | 55.59% | 44.41% |
|  |  |  |  |  |
| Skin reaction | My skin always burns in the sun, never tans and skin may peel. | 922 (10.1%) | 77.00% | 23.00% |
|  | My skin may tan but with difficulty in the sun and my skin may peel. | 1,463 (16.1%) | 82.80% | 17.20% |
|  | My skin burns moderately in the sun and tans gradually. | 2,496 (27.4%) | 77.80% | 22.20% |
|  | My skin rarely burns in the sun and tans easily. | 1,966 (21.6%) | 71.80% | 28.20% |
|  | My skin very rarely burns in the sun and tans very easily. | 1,112 (12.2%) | 58.60% | 41.40% |
|  | My skin never burns in the sun. | 1,143 (12.6%) | 34.70% | 65.40% |
|  |  |  |  |  |
| Fitzpatrick type | 1 | 482 (5.30%) | 71.20% | 28.80% |
|  | 2 | 729 (8.01%) | 74.20% | 25.80% |
|  | 3 | 807 (8.87%) | 74.50% | 25.50% |
|  | 4 | 956 (10.5%) | 74.10% | 25.90% |
|  | 5 | 1,423 (15.6%) | 70.30% | 29.70% |
|  | 6 | 1,243 (13.7%) | 70.60% | 29.40% |
|  | 7 | 906 (9.95%) | 70.30% | 29.70% |
|  | 8 | 738 (8.11%) | 66.00% | 34.00% |
|  | 9 | 818 (8.99%) | 65.60% | 34.40% |
|  | 10 | 590 (6.48%) | 61.00% | 39.00% |
|  | 11 | 233 (2.56%) | 55.40% | 44.60% |
|  | 12 | 177 (1.94%) | 59.30% | 40.70% |
|  |  |  |  |  |
| History of cancer | Yes | 1,328 (14.6%) | 80.60% | 19.40% |
|  | No | 6,892 (75.7%) | 67.90% | 32.10% |
|  | Don't know | 809 (8.9%) | 64.60% | 35.40% |
|  | Prefer not to answer | 73 (0.8%) | 67.10% | 32.10% |


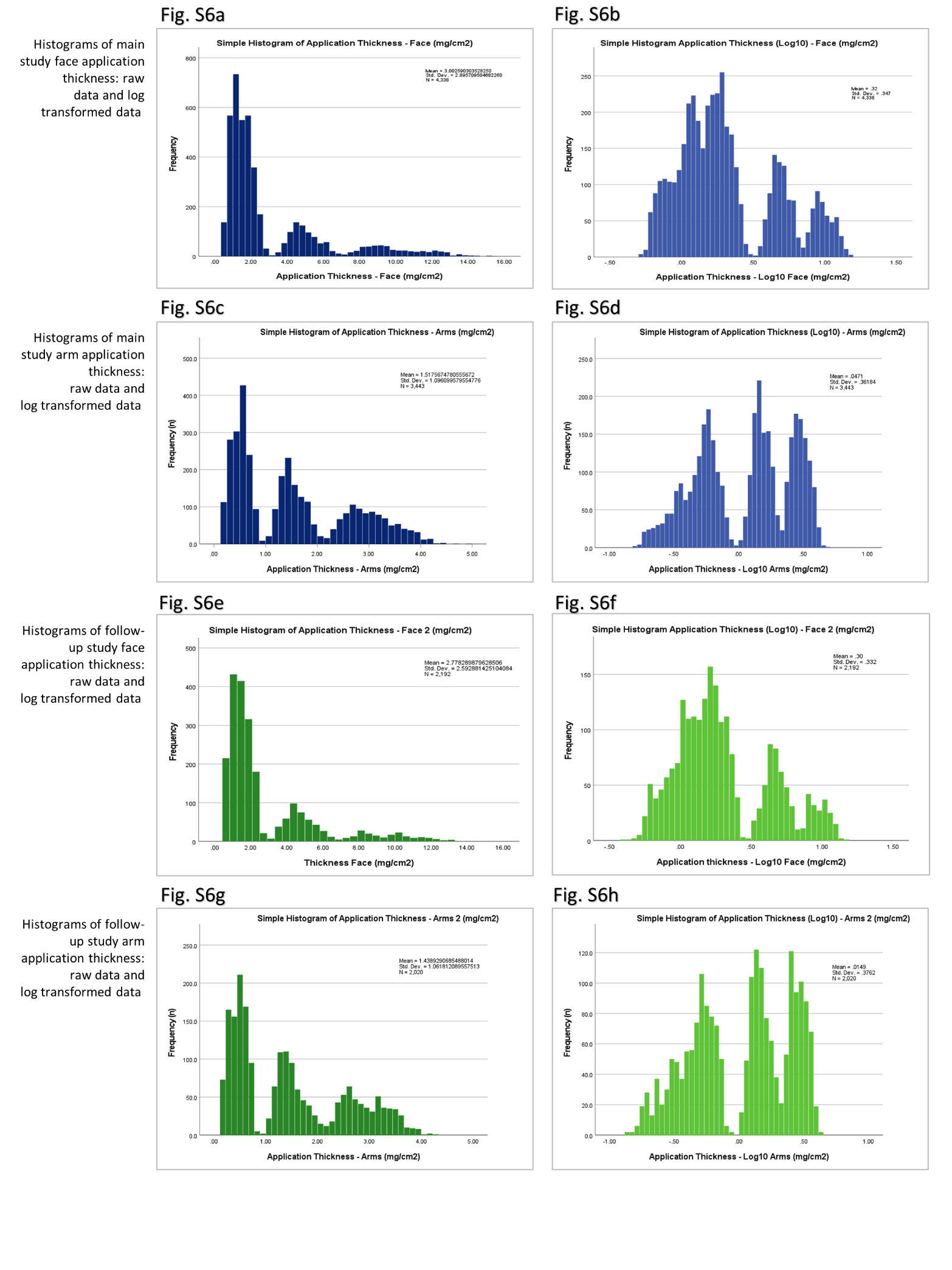
**Figure S3 Histograms of Application Thickness values – both untransformed and log10 transformed.**

**Table S4: Numeric assignments to independent variables**

**State of Residence**

Old Value New Value Value Label

Alabama 1 Alabama

Alaska 2 Alaska

Arizona 3 Arizona

Arkansas 4 Arkansas

California 5 California

Colorado 6 Colorado

Connecticut 7 Connecticut

Delaware 8 Delaware

District of Columbia 9 District of Columbia

Florida 10 Florida

Georgia 11 Georgia

Hawaii 12 Hawaii

Idaho 13 Idaho

Illinois 14 Illinois

Indiana 15 Indiana

Iowa 16 Iowa

Kansas 17 Kansas

Kentucky 18 Kentucky

Louisiana 19 Louisiana

Maine 20 Maine

Maryland 21 Maryland

Massachusetts 22 Massachusetts

Michigan 23 Michigan

Minnesota 24 Minnesota

Mississippi 25 Mississippi

Missouri 26 Missouri

Montana 27 Montana

Nebraska 28 Nebraska

Nevada 29 Nevada

New Hampshire 30 New Hampshire

New Jersey 31 New Jersey

New Mexico 32 New Mexico

New York 33 New York

North Carolina 34 North Carolina

North Dakota 35 North Dakota

Ohio 36 Ohio

Oklahoma 37 Oklahoma

Oregon 38 Oregon

Pennsylvania 39 Pennsylvania

Rhode Island 40 Rhode Island

South Carolina 41 South Carolina

South Dakota 42 South Dakota

Tennessee 43 Tennessee

Texas 44 Texas

Utah 45 Utah

Vermont 46 Vermont

Virginia 47 Virginia

Washington 48 Washington

West Virginia 49 West Virginia

Wisconsin 50 Wisconsin

Wyoming 51 Wyoming

**Gender Identity**

Old Value New Value Value Label

Female 1 Female

Gender variant/non-conforming 2 Gender variant/non-conforming

Male 3 Male

Prefer to self describe 4 Prefer to self describe

Transman (FtM) 5 Transman (FtM)

Transwoman (MtF) 6 Transwoman (MtF)

**Age Range**

Old Value New Value Value Label

18-24 1 18-24

25-34 2 25-34

35-44 3 35-44

45-54 4 45-54

55-64 5 55-64

65-70 6 65-70

**Ethnicity**

Old Value New Value Value Label

Asian or Pacific Islander 1 Asian or Pacific Islander

Bi-racial/Multiple ethnicities 2 Bi-racial/Multiple ethnicities

Black/African American 3 Black/African American

Hispanic 4 Hispanic

Indigenous 5 Indigenous

Other 6 Other

Prefer not to answer 7 Prefer not to answer

White/Caucasian 8 White/Caucasian

**Skin response to sun exposure**

Old value New Value

My skin always burns in the sun, never tans and skin may peel.  1

My skin may tan but with difficulty in the sun and my skin may peel.  2

My skin burns moderately in the sun and tans gradually.  3

My skin rarely burns in the sun and tans easily.  4

My skin very rarely burns in the sun and tans very easily.  5

My skin never burns in the sun.  6

**History of skin cancer**

Old value New Value

Yes 1

No, Don’t know, prefer not to answer or N/A 2

**Number of Children in the Household**

Old value New Value

Any numerical response 1

Adults only or Prefer not to answer 2

**Product SPF Range Used**

Old value New Value

SPF 15 or below 1

SPF 16-29 2

SPF 30-49 3

SPF 50+ 4

**SPF as regular part of skincare routine AND Use SPF outdoors more than 30mins**

Old value New Value

Agree strongly 1

Agree somewhat 2

Neither agree nor disagree 3

Disagree somewhat 4

Disagree strongly 5

**Residence in a warm or cold state**

| Old Value | New Value |
| --- | --- |
| Hawaii | 1 |
| Florida | 1 |
| Louisiana | 1 |
| Texas | 1 |
| Mississippi | 1 |
| Georgia | 1 |
| Alabama | 1 |
| South Carolina | 1 |
| Arkansas | 1 |
| Oklahoma | 1 |
| Arizona | 1 |
| North Carolina | 1 |
| Tennessee | 1 |
| California | 1 |
| Kentucky | 1 |
| Virginia | 1 |
| Missouri | 1 |
| Delaware | 1 |
| Kansas | 1 |
| Maryland | 1 |
| New Mexico | 1 |
| West Virginia | 1 |
| Illinois | 1 |
| New Jersey | 1 |
| Indiana | 1 |
| Ohio | 1 |
| Washington DC / District of Columbia | 1 |
| Nevada | 2 |
| Rhode Island | 2 |
| Nebraska | 2 |
| Pennsylvania | 2 |
| Connecticut | 2 |
| Utah | 2 |
| Iowa | 2 |
| Massachusetts | 2 |
| Oregon | 2 |
| Washington | 2 |
| Colorado | 2 |
| South Dakota | 2 |
| New York | 2 |
| Michigan | 2 |
| Idaho | 2 |
| New Hampshire | 2 |
| Wisconsin | 2 |
| Vermont | 2 |
| Montana | 2 |
| Wyoming | 2 |
| Maine | 2 |
| Minnesota | 2 |
| North Dakota | 2 |
| Alaska | 2 |

**Table S5: Logarithmic multiple variable regression analysis model results for sunscreen application thickness dataset**

| Model^a^ | R | R Square | Adjusted R Square | Standard Error of Estimate |
| --- | --- | --- | --- | --- |
| Face Application Thickness (log_10_) | 0.173 | 0.030 | 0.027 | 0.34169 |
| Arms Application Thickness (log_10_) | 0.256 | 0.066 | 0.062 | 0.35036 |

^a^ Predictors: state of residence, gender identity, age range, ethnicity, skin response to sun exposure, Fitzpatrick skin type, history of skin cancer, children in the household, product SPF range typically used, use of sunscreen when outdoors more than 30 minutes, the use of sunscreen in skincare routine, and residence in a warm or cold state

**Table S6: ANOVA table for sunscreen application thickness from an online survey of the US general population**

| Model |  | Sum of Squares | df | Mean Square | F | Significance |
| --- | --- | --- | --- | --- | --- | --- |
| Face Application Thickness (log_10_) | Regression | 15.563 | 12 | 1.297 | 11.109 | <0.001 |
|  | Residual | 504.248 | 4319 | 0.117 |  |  |
|  | Total | 519.812 | 4331 |  |  |  |
|  |  |  |  |  |  |  |
| Arms Application Thickness (log_10_) | Regression | 29.598 | 12 | 2.467 | 20.093 | <0.001 |
|  | Residual | 421.047 | 3430 | 0.123 |  |  |
|  | Total | 450.646 | 3442 |  |  |  |

**Figure S4: Respondents answers to the question “How much do you agree or disagree with the following statements:**

**References**

1. Bech-Thomsen N, Wulf HC. Sunbathers' application of sunscreen is probably inadequate to obtain the sun protection factor assigned to the preparation. Photodermatol Photoimmunol Photomed. 1992;9(6):242-4.

2. Neale R, Williams G, Green A. Application Patterns Among Participants Randomized to Daily Sunscreen Use in a Skin Cancer Prevention Trial. Archives of Dermatology. 2002;138(10):1319-25.

3. Gottlieb A, Bourget TD, Lowe NJ. Sunscreens: Effects of Amounts of Application of Sun Protection Factors. In: Lowe NJS, Nadim A; Pathak, Madhu A, editor. Sunscreens : development, evaluation, and regulatory aspects. 15. Second ed. New York: Marcel Dekker; 1997. p. 792.

4. Autier P, Boniol M, Severi G, Doré J-F, Research FTEOF, Group TOCMC-O. Quantity of sunscreen used by European students. British Journal of Dermatology. 2001;144(2):288-91.

5. Azurdia RMP, J.A; Diffey, B.L ; Rhodes, L.E. Sunscreen application by photosensitive patients is inadequate for protection. British Journal of Dermatology. 1999;140(2):255-8.

6. Azurdia RM, Pagliaro JA, Rhodes LE. Sunscreen application technique in photosensitive patients: a quantitative assessment of the effect of education. Photodermatology, Photoimmunology & Photomedicine. 2000;16(2):53-6.

7. Hart GC, Wright AL, Cameron RG. An Assessment of the Adequacy of Sunscreen Usage. Radiation Protection Dosimetry. 2000;91(1-3):275-8.

8. Lademann J, Schanzer S, Richter H, Pelchrzim RV, Zastrow L, Golz K, et al. Sunscreen application at the beach. Journal of cosmetic dermatology. 2004;3(2):62-8.

9. Szepietowski JC, Nowicka D, Reich A, Melon M. Application of sunscreen preparations among young Polish people. Journal of Cosmetic Dermatology. 2004;3(2):69-72.

10. Reich A, Harupa M, Bury M, Chrzaszcz J, Starczewska A. Application of sunscreen preparations: a need to change the regulations. Photodermatology, Photoimmunology & Photomedicine. 2009;25(5):242-4.

11. Bauer U, O’Brien DS, Kimlin MG. A New Method to Quantify the Application Thickness of Sunscreen on Skin. Photochemistry and Photobiology. 2010;86(6):1397-403.

12. Petersen B, Datta P, Philipsen PA, Wulf HC. Sunscreen use and failures – on site observations on a sun-holiday. Photochemical & Photobiological Sciences. 2013;12(1):190-6.

13. De Villa D, da Silva Nagatomi AR, Paese K, Guterres S, Cestari TF. Reapplication Improves the Amount of Sunscreen, not its Regularity, Under Real Life Conditions. Photochemistry and photobiology. 2011;87(2):457-60.

14. Narbutt J, Philipsen PA, Harrison GI, Morgan KA, Lawrence KP, Baczynska KA, et al. Sunscreen applied at ≥ 2 mg cm−2 during a sunny holiday prevents erythema, a biomarker of ultraviolet radiation‐induced DNA damage and suppression of acquired immunity. British journal of dermatology (1951). 2019;180(3):604-14.

15. Teramura T, Mizuno M, Asano H, Naito N, Arakane K, Miyachi Y. Relationship between sun-protection factor and application thickness in high-performance sunscreen: double application of sunscreen is recommended. Clinical and experimental dermatology. 2012;37(8):904-8.

16. Pissavini M, Doucet O, Diffey B. A novel proposal for labelling sunscreens based on compliance and performance. International journal of cosmetic science. 2013;35(5):510-4.

17. Novick R, Anderson G, Miller E, Allgeier D, Unice K. Factors that influence sunscreen application thickness and potential preservative exposure. Photodermatology, photoimmunology & photomedicine. 2015;31(4):212-23.

18. Ficheux AS, Chevillotte G, Wesolek N, Morisset T, Dornic N, Bernard A, et al. Consumption of cosmetic products by the French population second part: Amount data. Food and Chemical Toxicology. 2016;90:130-41.

19. Heerfordt IM, Torsnes LR, Philipsen PA, Wulf HC. Photoprotection by sunscreen depends on time spent on application. Photodermatology, Photoimmunology & Photomedicine. 2018;34(2):117-21.

20. Heerfordt IM, Philipsen PA, Larsen BØ, Wulf HC. Long-term trend in sunscreen use among beachgoers in Denmark. Acta dermato-venereologica. 2017;97(10):1202-5.

21. Harben A, Robinson S, de la Fuente J, Bix L. The Role of Dispensing Device and Label Warnings on Dosing for Sunscreen Application: A Randomized Trial. Health Education & Behavior. 2019;47(1):143-52.

22. Heerfordt IM, Torsnes LR, Philipsen PA, Wulf HC. Sunscreen use optimized by two consecutive applications. PloS one. 2018;13(3):e0193916-e.

23. Heerfordt IM, Philipsen PA, Wulf HC. A Handful of Sunscreen for Whole-Body Application. In: Reichrath J, editor. Sunlight, Vitamin D and Skin Cancer. Cham: Springer International Publishing; 2020. p. 381-5.

24. Dang J, Reserva J, Tung-Hahn E, Vasicek B, Krol C, Adams W, et al. Sunscreen application technique amongst patients with a history of skin cancer. Archives of Dermatological Research. 2020;312(10):739-46.
